# Toward “super-scintillation” with nanomaterials and nanophotonics

**DOI:** 10.1515/nanoph-2023-0946

**Published:** 2024-04-15

**Authors:** Hamish Carr Delgado, Parivash Moradifar, Garry Chinn, Craig S. Levin, Jennifer A. Dionne

**Affiliations:** Department of Materials Science and Engineering, 6429 Stanford University, Stanford, CA 94305, USA; Department of Radiology, 6429 Stanford University, Stanford, CA, 94305, USA

**Keywords:** high-energy radiation, nanophotonic scintillation, super scintillator, nanoscintillating materials

## Abstract

Following the discovery of X-rays, scintillators are commonly used as high-energy radiation sensors in diagnostic medical imaging, high-energy physics, astrophysics, environmental radiation monitoring, and security inspections. Conventional scintillators face intrinsic limitations including a low extraction efficiency of scintillated light and a low emission rate, leading to efficiencies that are less than 10 % for commercial scintillators. Overcoming these limitations will require new materials including scintillating nanomaterials (“nanoscintillators”), as well as new photonic approaches that increase the efficiency of the scintillation process, increase the emission rate of materials, and control the directivity of the scintillated light. In this perspective, we describe emerging nanoscintillating materials and three nanophotonic platforms: (i) plasmonic nanoresonators, (ii) photonic crystals, and (iii) high-Q metasurfaces that could enable high performance scintillators. We further discuss how a combination of nanoscintillators and photonic structures can yield a “super scintillator” enabling ultimate spatio-temporal resolution while enabling a significant boost in the extracted scintillation emission.

## 1 Introduction

Scintillation is the spontaneous conversion of high-energy ionizing radiation, as well as charged and neutral particles (e.g., α-, β-, γ-, X‐rays, electrons and neutrons), into light. Upon the incidence of high-energy radiation, a scintillating material absorbs, converts, and re-emits numerous low-energy detectable photons in the ultraviolet-to-visible (UV–Vis) regime. A scintillator, therefore, acts as a wavelength shifter for high-energy radiation and charged particles [1], [2]. The first notable use of scintillator devices dates back to 1903 when Crookes and Rutherford used silver-activated zinc sulfide (ZnS: Ag) to detect the luminescent “flashes of light,” from invisible α-particle radiation during Rutherford’s gold foil experiments. They used a simple device they called a “spinthariscope,” which combined a ZnS screen and a microscope to view the scintillated light produced during the interaction of the α-particles with the zinc sulfide screen [3]–[5].

Nowadays, scintillation underlies a myriad of technologies, spanning diagnostic medical imaging, radiation therapy, security inspection tools, environmental radiation monitoring, high energy astrophysics, and interplanetary space missions. Scintillators play a prominent role in bioimaging and biomedicine [6]–[8] including gamma cameras, single photon emission computed tomography (SPECT), positron emission tomography (PET), X-ray induced photo dynamic therapy (X-PDT) [9]–[11], and intraoperative imaging enabling radioguided surgical procedures [12]. In gamma cameras, SPECT and PET, scintillators are the major detection unit to record and spatially correlate high energy photons to produce images of the radionuclide distribution. For PET, precise timing is an important scintillator property since annihilation photons (γ-rays) are produced in pairs by positron–electron annihilation, and the differential arrival times of the detected annihilation photons can be used for time-of-flight imaging to improve the reconstructed image signal-to-noise ratio (SNR). In radiation therapy such as X-PDT, X-rays with unrestricted tissue penetration depth are used as an excitation source in conjunction with nanoscintillators as energy mediators and photosensitizers for targeted and noninvasive deep tumor therapy [9], [10], [13]. Additionally, nanoscintillators are emerging as promising candidates for noninvasive *in vivo* optogenetics applications enabling neuromodulation and stimulation of light sensitive proteins in brains to advance our understanding of the neural pathways and control synaptic functions in brain [14], [15]. In homeland security, scintillators are used for screening in border crossings such as airports and seaports for baggage, cargo, and vehicle inspections to prevent illegal traffic, concealed weapons, prohibited and fissile materials. In industrial applications, scintillators are used for nondestructive inspection of structures, such as airplane turbine blades, oil pipes, and concrete beams, to assess their integrity through techniques like X-ray computed tomography, which can produce high-resolution tomographs of the structures to identify internal defects such as voids and cracks [16]–[18]. In environmental monitoring, scintillators are used as dosimeters to evaluate the risk associated with natural or artificial radionuclides to identify the type and level of activity. Finally, in space missions, γ-ray detectors are used for detecting cosmic rays and gamma-ray bursts [1], 19]

**Figure 1:**
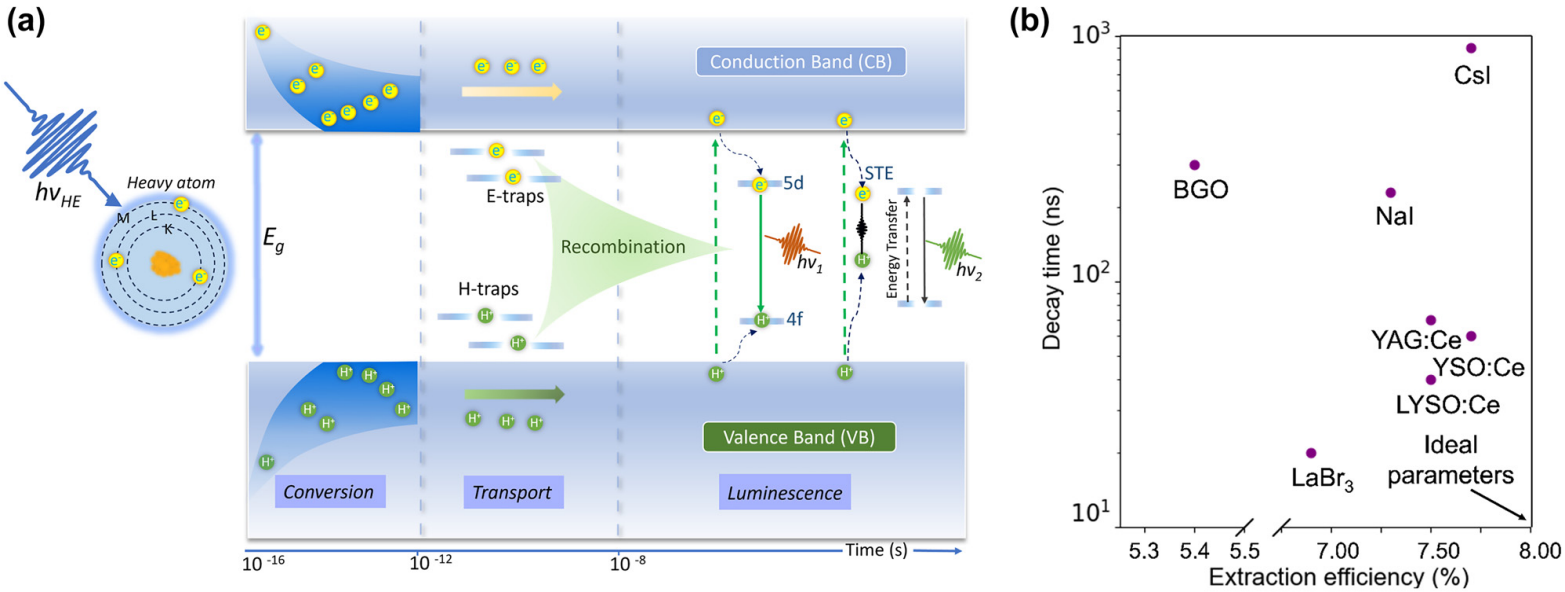
Overview of scintillation process and performance metrics (extraction efficiency vs. decay lifetime) of common inorganic scinitllators. (a) Schematic of the three stages governing the emission process during a scintillation event: (i) conversion, (ii) energy transport, and (iii) recombination and luminescence emission. (b) Quantitative comparison of decay time and light extraction efficiency for commonly used scintillation materials. The extraction efficiency was determined using the 1/4*n* ^2^ formula [28].

Scintillation mechanism is a multi-stage process, which consists of three major stages or subprocesses. The three stages governing the emission process during a scintillation event are (i) conversion, (ii) energy transfer or transport, and (iii) recombination and luminescence emission, as shown in Figure 1(a) [20]. Several scintillation mechanisms have been identified including (i) emission from doping centers, (ii) self-activated or excitonic emission, and (iii) cross-luminescence also referred as core-valence emission or Auger-free emission [21], [22]. Scintillation materials comprise any form of gas, liquid, and solid as well as organic and inorganic materials. Additionally, scintillators are categorized as intrinsic or extrinsic/activated. Excitonic emission and cross-luminescence are examples of intrinsic scintillation mechanisms. BaF_2_ is an example of an intrinsic scintillator with two radiative decay components. The slow radiative component with decay lifetime of 620 ns originates from excitonic emission of self-trapped excitons (STEs). While an ultra-fast decay component of sub 1 ns (∼600 ps) with emission wavelength of approximately 250 nm arises from cross-luminescence. Cross-luminescence originates from deep electron–hole recombination and is observed in other fluoride host lattices such as CsF, BaLu_2_F_8_ [23], [24]. Furthermore, self-activated (i.e. excitonic) scintillation is the dominant mechanism in Bi_4_Ge_3_O_12_, CeF_3_, and LuTaO_4_. Extrinsic scintillation, also referred to as activated scintillation or impurity induced scintillation, is the main process behind the majority of inorganic scintillators in which dopants or impurities are added as emission centers to the host material. Thallium, sodium, cerium, and europium are examples of commonly used activators, and NaI:Tl^+^, CsI:Na^+^, CsI:Tl^+^, and CaF_2_:Eu^2+^ are examples of extrinsic scintillator materials. NaI:Tl^+^ and CsI:Tl^+^ are among the brightest scintillators with scintillation yield of ∼20 photons/keV and 40–60 photons/keV respectively [21], [25]–[27].

Some outstanding challenges with commercial inorganic scintillators are the low extraction efficiency of scintillated light and low emission rate of the materials. The first challenge originates from the high stopping power required for high-energy ionizing radiation, which is accompanied by a large refractive index of the medium at the emitted wavelength. Given that most scintillating materials re-emit light isotropically, only light traveling at angles smaller than the critical angle for total internal reflection can be extracted from the material. For example, Bi_4_Ge_3_O_12_ (BGO), a common scintillator, has a light extraction efficiency of 5.4 % due to these losses, and most commonly used scintillators have efficiencies below 10 % (Figure 1(b)) [29]. On the other hand, the emission rate limits the temporal resolution of imaging and is typically intrinsic to the scintillating material [30].

The quest for high-performance scintillators is of high interest to both the research and industrial communities. Desired properties include linear energy proportionality, high light yield, superior timing performance, ultrafast rise- and -decay lifetime, and enhanced light extraction efficiency [24]. Notably, light yield and its linear proportionality are important for energy resolution and contribute to the time resolution of the scintillator. The rise time is attributed to the transfer from vibrational modes to luminescent centers, and the decay time is dependent on the emission and quenching of luminescent centers. Both rise and decay time contribute to the timing resolution. Additionally, other materials properties such as density, effective Z, radiation hardness, refractive index for effecient coupling to the photodetector, and optical transparency for effecient transmission of photons to photodetectors should taken into consideration for optimizing the performance metrics. In particular, designing new scintillators with ultraprecise temporal resolution will open new possibilities for time-of-flight (TOF) applications, such as reconstruction-free time-of-flight positron emission tomography (TOF-PET). In TOF-PET, the signal-to-noise ratio of the reconstructed images depends on the coincidence time resolution and affects the image quality and lesion detection accuracy. Ultraprecise timing will also allow quality imaging at reduced injected dose. In other words, improved temporal resolution in PET will enable safer and more precise disease detection and monitoring [24], [27], [31], [32].

**Figure 2:**
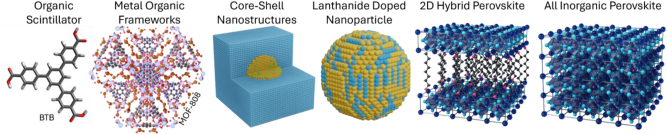
Schematics of a number nanoscintillating building blocks including but not limited to, organic scintillators (BTB), metal–organic frameworks (zirconium-based MOF-808), rare-earth doped nanoparticle, various oxide and sulfide core–shell nanostructure, 2D hybrid perovskites, and all inorganic perovskites.

A solution to these challenges will be two-fold: developing new nanomaterials for scintillation (“nanoscintillators”), as well as new photonic approaches to control both the high-energy absorption and optical emission from the scintillators. Regarding nanoscintillators, radioluminescent nanostructures have emerged as promising building blocks, offering various tuning knobs ranging from composition, morphology to controlling the symmetry, and anisotropy of individual building blocks to overcome the intrinsic limitations of bulk scintillators. Various degrees of freedom in design, fabrication, and macroscale assembly architecture allow to not only tailor the emission wavelength over a broad spectral range (deep-UV to NIR) but also for manipulation of emission dynamics and controlling the radiative processes involved. Low-dimensional materials have emerged as promising building blocks for scintillation applications, covering a broad range of organic, inorganic, and hybrid structures. Figure 2 showcases examples of organic and molecular nanostructures (benzene-1,3,5-trisbenzoate (BTB)), inorganic nanostructures such as rare-earth doped nanoparticles, and all-inorganic perovskites as well as hybrid scintillating nanostructures such as metal–organic frameworks such as Zr-based MOF-808, and hybrid perovskites (Figure 2). Following the first report of scintillation response in quantum dots (QDs) by Létant and Wang in 2006 [33], several other emerging materials platforms, low-dimensional platforms, have been proposed and evaluated including metal halide perovskites [34], [35], all inorganic perovskites [36], organic–inorganic layered perovskites, scintillating nanotubes [37], [38], lanthanide-doped nanoparticles [7], [10], and metal–organic frameworks [39], [40]. In addition to rare-earth doped nanoscintillators, various types of oxides [41], [42], halides [43], sulfides [44], and oxysulfides scintillating nanostructures [45] with varying refractive index have been designed and evaluated offering a tunable range of scintillation wavelength, morphology, decay lifetime, and radiation hardness. Despite a plethora of advantages including outstanding spectral range tunability and flexibility of design, nanoscintillators can suffer from low quantum yield originating from thermal quenching and large self-absorption compared to their bulk counterparts. Integrating these nanomaterials into well-established nanophotonic platforms promises to overcome challenges, as we describe in this perspective.

In parallel, nanophotonic platforms have emerged to sculpt the interaction of high-energy radiation with both bulk and nanoscintillators. Nanophotonic platforms can boost the performance of scintillators by increasing the efficiency of the scintillation process, increasing the emission rate of materials, and controlling the directivity of the scintillated light. By combining these approaches, it is possible to improve the light extraction efficiency and spatio-temporal resolution of scintillation emission. This perspective discusses various nanophotonic scintillation platforms that can be used independently or in conjunction with nanoscintillating building blocks to achieve high spatio-temporal resolution and a significant boost in the extracted scintillation lights. We also highlight how hybrid platforms consisting of nanoscintillator building blocks and nanophotonic platforms can reinforce one another, creating a “super scintillator” that is more than the sum of its parts.

## 2 Enhancing and shaping scintillating emission with nanophotonics

Nanoresonators, such as plasmonic antennas and photonic crystals, enhance the rate of spontaneous emission of various emitters through an increase in the local density of photonic states (LDOS) [46]–[49]. Recent seminal work by Roques-Carmes et al. presented a general framework for scintillation in nanophotonics [50]. This rational basis for the design of nanophotonic structures to enhance scintillating efficiency is based on Lorentz reciprocity of Maxwell’s equations, which allows calculating the scintillation emission of a given nanophotonic structure by calculating its absorption of plane waves. Furthermore, this framework demonstrates that scintillation is enhanced for wavelengths at which the electromagnetic fields are increased in the nanophotonic structure over the effective volume of scintillation.

**Figure 3:**
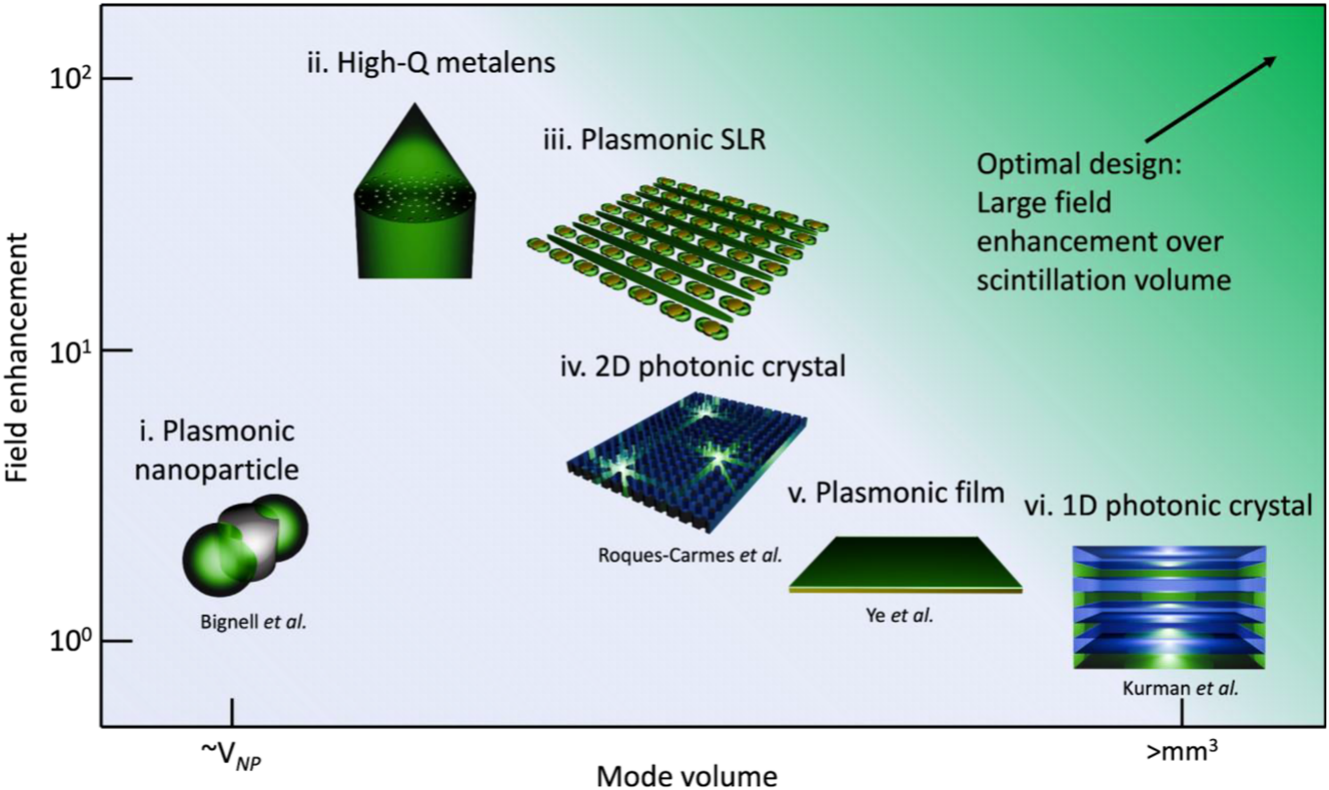
Schematic of various nanophotonic platforms for scintillation enhancement. (i) Plasmonic nanoparticles (study from Bignell et al.) provide localized field enhancement in small sub-wavelength mode volumes approximately the volume of the nanoparticle [55]. Such platforms with small mode volumes can result in scintillation enhancement provided many of them are distributed over large areas. (ii) High-Q metalenses are promising for focusing scintillated light while enhancing scintillation efficiency by coupling to high-Q resonances. (iii) Plasmonic SLRs provide higher field enhancements than isolated nanoparticles, with mode volumes that extend over the entire area of the array, while providing directional emission to the 0th diffraction order. (iv) 2D photonic crystals can boost emission intensity through field enhancements, as well as provide directional emission in the out-of-plane direction. These can be fabricated atop scintillating crystals, or by patterning the scintillating material itself (field enhancement value from Roques-Carmes et al.). (v) Unpatterned plasmonic films are able to provide moderate field enhancements while extending through large areas over the entire surface of conventional scintillators (field enhancement value from Ye et al.). (vi) 1D photonic crystals (field enhancement value from Kurman et al.) can provide enhancement in the emission intensity and directivity through low-Q resonances that extend through the entire volume of the photonic crystal. These can be made by alternating layers of scintillating materials with common dielectrics. With a sufficient number of layers, the enhancement volume can exceed mm^3^. For patterned structures, such as high-Q metalenses, plasmonic SLRs, and 2D photonic crystals, the mode volume will be dependent on the footprint of the nanophotonic structure.

One useful metric to design nanophotonic scintillators is the integral of the field enhancement over the scintillating volume in the nanophotonic structure. While there are early demonstration of coupling nanophotonic platforms to conventional scintillating materials [50]–[54], the marriage of scintillation and nanophotonics is still a nascent field, and we believe that unexplored combinations of these techniques will give rise to a new generation of more efficient nanoscale scintillators. Figure 3 highlights some of the pioneering work in the field, spanning plasmonics, photonic crystals, and metasurfaces for scintillation.

### 2.1 Plasmonic resonators

Plasmonics involves the interaction of light with the collective oscillation of conduction electrons in metals, leading to significant electromagnetic field enhancements and field localization in small, generally nanoscale volumes. Several types of plasmonic resonances are achievable, including localized surface plasmon resonances (LSPRs), propagating surface plasmon polaritons (PSPPs), and surface lattice resonances (SLRs) [56], [57]. Beyond their field enhancement, plasmonic metals have a high atomic number, enabling a high stopping power for the incident ionizing radiation.

In 2013, Bignell et al. reported 13 % enhancement in scintillation intensity, using covalent binding of a solution-phase organic scintillator (9-aminoacridine) to plasmonic silver-silica core-shell nanoparticles [53]. More recently, metallic thin films have been incorporated into scintillating perovskite materials, achieving scintillation yield of 88 photons/keV, an over 2 fold yield enhancement, through the Purcell effect from propagating surface plasmons at the metal–dielectric interface, as reported by Ye et al. [52]. Additionally, this work predicts over 10-fold enhancement in the emission rate of the material. This approach provides significant enhancement while maintaining a planar geometry, which is desirable in terms of ease of fabrication. However, the scintillation enhancement that these platforms can achieve remains relatively low, owing to low quality factor resonances. Patterning of the plasmonic films could provide further yield enhancement and better directivity of the scintillated light.

Surface lattice resonances (SLRs) are comprised of a periodic array of nanoparticles, collectively interacting to produce resonances with narrower spectral widths and higher quality factors than isolated nanoparticles, while supporting extended photonic modes over large areas [56], [58], [59]. SLRs, with tunable properties based on geometric parameters, have achieved quality factors exceeding 2000 [58], [59]. Coupling SLRs to various emitters enhances their emission rate and directivity but necessitates close index matching for high-quality factor resonances in inhomogeneous environments. Foundational work by Liu et al. has reported the coupling of organic scintillators to SLRs of silver nanoparticles, resulting in an overall 1.81-fold enhancement of scintillation emission from the plastic scintillating film on on a silicon substrate [58].

### 2.2 Photonic crystals

Complementing plasmonic structures for scintillation are photonic crystals. Photonic crystals are materials with a periodicity in their dielectric constant at scales comparable to the wavelength of light. The photonic crystal periodicity gives rise to photonic bandgaps or “forbidden frequencies,” at which photons cannot propagate through the material [60], [61]. While plasmonics involves hybrid photon–electron oscillations, which afford high control of the nearfield, photonic crystals are more general periodic arrangements of dielectric media, which determine the optical band structure of the material, reflected in the farfield.

Some of the earliest work with photonic crystals and scintillators has focused on improving light extraction from scintillators. Thin photonic crystal layers placed at the interface between the scintillator and photodetector are used to grade the index of refraction from the scintillator to the photodetector window. Fabricated scintillators have been experimentally measured and shown to increase the light collection efficiency by over 40 % [62], [63]. Going beyond the scintillator–photodetector interface, one-dimensional (1D) photonic crystals for scintillating applications have been reported by the pioneering work of Kurman et al. [51]. Here, a 1D photonic crystal consisting of alternating layers of SiO_2_ with a conventional scintillating material, europium- and bismuth-doped lutetium(III) oxide (Lu_2_O_3_), resulted in a 50 % enhancement in emission rate and an 80 % enhancement in light yield of the scintillating material, acheiving an overall light yield of 26,000 photons/MeV. Calculations suggest that even higher efficiencies are possible, with a fivefold enhancement of detectable photons while maintaining directional emission [51], [64].

Two-dimensional (2D) photonic crystals can efficiently enhance scintillation intensity and emission rates by leveraging the high field enhancements that are achievable. They also control light extraction directionality due to the in-plane photonic bandgap, redirecting emitted light exclusively along the out-of-plane dimension for improved directional emission [65], [66]. Seminal work by Kronberger et al. and Knapitsch et al. predicted over twofold enhancement in the light extraction and improved directional emission from conventional scintillators by patterning 2D photonic crystals on top of them [54], [67]. Recently, a common scintillating material, YAG:Ce, was patterned into a 2D photonic crystal by etching periodic air holes in a square lattice pattern, as reported by Roques-Carmes et al. [50]. The photonic crystal scintillator was reported to exhibit a 9.3-fold enhancement in emission intensity compared to the unpatterned region of the scintillator crystal.

### 2.3 Metasurfaces

Metasurfaces enable precise control of light propagation on small scales. Metasurfaces are made of metallic or dielectric constituents, i.e., meta-atoms, which act as antennas to control the properties of light. The ability to control both the nearfield and the farfield radiation patterns simultaneously makes metasurfaces ideal platforms for scintillation enhancement, combining the advantages afforded by photonic crystals and plasmon polaritons in an intergrated platform. By tuning the geometry of the meta-atoms, it is possible to construct wavefront shaping platforms, such as beam steerers and lenses, in a compact, two-dimensional platform. Despite the ability of plasmonic metasurfaces to confine light in deep subwavelength volumes, metasurfaces suffer from large intrinsic losses, which can be overcome by all-dielectric metasurfaces.

Metalenses enable focusing of light in a compact, lightweight platform that can be easily integrated into most imaging and light detection systems. In addition to performing the same functions as conventional refractive lenses, metalenses can offer high efficiency, high numerical aperture, aberration correction, and multifunctionality in an integrated platform [68]–[70]. In the context of scintillation, metalenses can be beneficial by collimating scintillation emission to a photodetector, thereby increasing the efficiency of light collection and minimizing the required high-energy radiation dose to achieve high-resolution imaging. A recent study by Uenoyama et al. demonstrated a decrease in the coincidence time resolution – an important performance parameter for the accuracy of clinical PET detectors [71], [72] – of 54.33 ps by collimating scintillation emission by integrating a HfO_2_ metalens into a silicon photomultiplier, compared to a design without the metalens [73].

Recently, dielectric metasurfaces supporting high-Q resonances have been demonstrated, enabling precise control of the outgoing wavefront of light while having nearfield enhancement [74]. Wavefront shaping can be easily achieved by both dielectric and metallic metasurfaces by phase tuning of meta-atoms. On the other hand, high-Q resonances can be supported by metallic metasurfaces (SLRs) at the expense of the arbitrary wavefront shaping ability of the meta-atoms, given the coherent coupling required for these lattice resonances, which necessitates identical geometry from all the meta-atoms. Dielectric metasurfaces, on the other hand, are excellent candidates for achieving wavefront shaping while supporting high-Q resonances. Examples of high-Q wavefront shaping metasurfaces can be found in high-Q beamsteering metasurfaces and high-Q metalenses [61]–[75].

As a subclass of high-Q metasurfaces, high-Q metalenses are of particular interest for scintillation applications given their ability to focus specific narrowband wavelengths, while supporting resonances with very large field enhancements. High-Q metalenses provide increased functionality compared to nonresonant metalenses, given their simultaneous ability to focus and collimate light, while enhancing the emission rate and intensity through increased LDOS. High-Q silicon metalenses have been reported exhibiting quality factors exceeding 1000 using guided mode resonances on silicon nanoantennas [75], [76]. High-Q silicon metalenses enable simultaneous control of the resonant wavelength as well as resonance quality factor through tailoring the geometry of the nanoantennas, making high_Q metalenses a promising platform for effeciently collimating the scintillation emission at given wavelengths. Furthermore, high-Q metalenses support guided mode resonances with photonic mode volumes that extend through the length of the nanoantennas, making high-Q metalenses promising candidates for scintillation enhancement over extended areas. The enhanced emission and strong collimation of scintillated light into a photodetector could enable the use of lower radiation doses in PET and other applications where minimizing radiation dose and radiation damage is desirable.

## 3 Conclusions and outlook

The realization of more efficient scintillators through nanophotonic enhancement can enable several impactful technologies and applications, ranging from low-dose and safer diagnostic imaging, ultra-sensitive environmental radiation monitoring systems and nuclear cameras, micro- and nano-CT scanners with single-molecule resolution, to sensors for dark matter in astrophysics research and interplanetary space missions. Here we have outlined the challenges with conventional bulk scintillators, emphasizing the need for designing versatile and high-performance scintillation platforms that provide enhanced temporal, emission rate, and light extraction efficiency. We have introduced various different types of nanoscintillating building blocks as well as three nanophotonic platforms for scintillation, (i) plasmonic nanoresonators, (ii) photonic crystals, and (iii) high-Q metasurfaces, and several nanoscintillating building blocks to achieve high spatio-temporal resolution and a significant boost in the extracted scintillation light.

Looking ahead, integration of plasmonic SLRs atop scintillating materials is a promising avenue for enhanced scintillation. These SLR structures offer efficient coupling of light into strong plasmonic modes extended over extended areas, and are ideally suited for large enhancements in scintillation intensity and emission rate enhancement. Furthermore, the large atomic number of plasmonic materials can provide additional stopping power for high energy radiation. We also note that 3D photonic crystals coupled to scintillators remain unrealized. We believe that this platform offers untapped potential for controlling the propagation of scintillated light, while allowing for large field enhancements in extended volumes [77], [78]. Additionally, a 3D multilayered structure will be beneficial for increasing the stopping power over larger volumes of the photonic crystal, compared to 2D structures. While these benefits come at the expense of challenging fabrication, a promising approach consists of structuring nanoscintillators into larger 3D structures, potentially through the use of nanoimprint lithography and additive manufacturing [79], [80]. Finally, we envision that integrating high-Q metalenses into scintillators will greatly increase the collimation and extraction efficiencies of scintillation yield. In addition, high-Q beamsteering metasurfaces can be designed to redirect the enhanced scintillation emission to specific diffraction orders [74]. Such devices can be used in diffractive neural networks to perform sophisticated computations, especially in the emergent field of X-ray quantum optics [81], [82].

Combining low-dimensional engineered building blocks, e.g., nanoscintillators, and nanophotonic platforms could yield a “super scintillator” that offers ultimate performance. Integrating nanoscintillators into nanophotonic platforms can be accomplished in several ways, including drop casting and spin coating nanoparticles onto nanophotonic structures [83], [84], direct patterning of the 2D materials by lithography [85], and through molecular self-assembly of the nanoscintillators into 3D photonic crystals [86]. While there is no one “ideal scintillator,” hybrid approaches will allow tailoring the scintillation properties to create the ideal scintillator for any given existing application as well as fulfilling emerging applications. Outstanding conversion efficiency and ultrafast decay and rise time are examples of an ideal scintillator that will open new frontiers in the future of precision health and space explorations. In diagnostics, such a super-scintillator could enable ultra-low radiation dose PET imaging, to provide such scans to vulnerable patients (e.g., children, pregnant women), reducing potential damage to the fetus. It could also dramatically boost the SNR of PET enabling the earlier cancer detection and improve the success rate of oligometastatic treatment, an early phase of curable metastatic disease. Reducing the radiation dose will also enable more frequent monitoring of treatment and with greater accuracy. In materials and biological research, improved X-ray imaging can yield high-resolution, potentially nondestructive imaging of single cells and proteins [87], [88]. In particular, developing ultra-fast scintillators with sub-picosecond lifetime will be of major interest in hard X-ray imaging in GHZ regime with a prominent application in high-repetition X-ray free electron laser facilities (XFEL). Additionally, in environmental monitoring, better scintillators could be used for ultrasensitive, real-time detection of radioactive waste – potentially down to the single radionuclide level. Lastly, next generation scintillator detectors with ultra-high energy resolution will aid in the detection and identification of dark matter signatures in space exploration, by measuring the energy of the interaction between weakly interacting massive particles, and a normal nucleus, which is ∼100 keV [89]. Though nanophotonic scintillation is nascent, with the rapid development of new nanomaterials combined with tailored nanophotonic platforms, we envision a bright future for this highly out-of-equilibrium process with advances spanning across precision health, ultra-sensitive and targeted security inspections, and environmental radiation monitoring with extremely low detection limit.
